# Swiss trade during the COVID-19 pandemic: an early appraisal

**DOI:** 10.1186/s41937-020-00069-3

**Published:** 2020-12-28

**Authors:** Konstantin Büchel, Stefan Legge, Vincent Pochon, Philipp Wegmüller

**Affiliations:** 1 Department of Economics & Center for Regional Economic Development (CRED), University of Bern, Schanzeneckstrasse 1, Bern, CH-3001 Switzerland; 2 Department of Economics, SIAW Institute, University of St.Gallen, Bodanstrasse 8, St. Gallen, CH-9000 Switzerland; 3 State Secretariat for Economic Affairs (SECO), Holzikofenweg 36, Bern, CH-3003 Switzerland

**Keywords:** Covid-19, Trade, Switzerland, E32, F14, H12, I18

## Abstract

This study uses trade data from Switzerland’s Federal Customs Administration to examine the impact of Covid-19 on international goods trade between January and July 2020. We show that Swiss trade during that period fell by 11% compared to 2019 and that the contraction following the “Federal Lockdown” in mid-March was considerably steeper than the Swiss trade collapse in the aftermath of the Lehman Brothers bankruptcy in September 2008. Examining cross-country variation in Covid-19 cases, the stringency of containment measures, and Swiss trade flows, we document that the pandemic adversely affected both the demand and supply side of foreign trade, while trade restrictions and exchange rate fluctuations played no major role behind the rapid decline of Swiss trade in the first half of 2020.

## Introduction

Cross-border trade of goods and services is one of the primary sources of economic prosperity and of particular importance for small open economies like Switzerland. The COVID-19 pandemic imposes barriers to international economic exchange as potentially no other event in the recent past.^1^ Early into the pandemic, Baldwin (2020) hypothesized that the COVID-19 induced decline in trade might even surpass the contraction in the aftermath of the financial crisis in 2008, since the spread of the virus and the widely adopted countermeasures simultaneously inflict a heavy burden on both the supply and demand side.

This paper provides an early characterization of Swiss trade during COVID-19 based on official Swiss trade data at the product and country level. To put recent developments into perspective, we compare exports and imports since January 2020 with trade flows during the global recession that was ultimately triggered by the bankruptcy of Lehman Brothers in September 2008. We further discuss several channels that help understand potential drivers behind the documented patterns. In particular, we focus on COVID-19 induced demand and supply side dynamics by exploiting cross-product and cross-trading-partner variation. We also appraise additional factors such as international trade policy and exchange rate movements.

We show that on the outset of 2020, both the value of exported and imported goods hovered around similar levels as in 2019. This dramatically changed in mid-March, when the spread of COVID-19 accelerated and the Swiss Federal Council announced far-reaching containment measures. Until mid-year in June 2020, the accumulated value of trade fell by roughly 10% compared to 2019, and the contraction would have been considerably fiercer absent the strong performance of the chemical and pharmaceutical industry. Comparing the 2020 trade contraction to the losses during the global financial crisis, we illustrate that the COVID-19 triggered downward spiral occurred much faster and was substantially steeper. However, unlike after the insolvency of Lehman Brothers, first signs of recovery emerged already within 3 months.

Examining the drivers behind Switzerland’s foreign trade collapse, we show that the export losses coincided with a sizeable deterioration of consumer confidence. Moreover, exports during the first two quarters of 2020 are robustly correlated with the trading partner-specific COVID-19 infection rates, but almost orthogonal to the stringency of country-specific containment measures. Trading partner-specific Swiss import dynamics, on the other hand, are correlated with both the stringency of governmental containment policies as well as—albeit weaker—with COVID-19 infection rates. Overall, the data lends little support to the narrative that the costly economic fall-out of COVID-19 should be *primarily* attributed to the unprecedented public health policies; yet, we find some evidence that stringent containment measures adopted by trading partners imposed costly barriers to the foreign producers of Swiss imports. Finally, we document that neither protectionist trade measures nor exchange rate movements in 2020 played a major role behind the rapid decline in Swiss trade volumes.

Our work contributes to a growing economic literature that aims to shed a light on the mechanics and consequences of the COVID-19 pandemic.^2^ Previous articles on potential trade effects of COVID-19 were either based on simulations (e.g., Benz, Gonazles, & Mourougane, 2020; Maliszewska, Mattoo, & Van Der Mensbrugghe, 2020), empirical analysis of related events such as the SARS outbreak in 2003 (Fernandes & Tang, 2020), or a combination of descriptive historical comparisons and economic reasoning (Baldwin, 2020; Gruszczynski, 2020). Using a rich data set covering Swiss trade until July 2020, we can provide an early characterization of trade dynamics during COVID-19. Our work is also inspired by prior research on what Baldwin (2009) called the *Great Trade Collapse*—the decline in international trade following the global financial crisis that culminated in the bankruptcy of Lehman Brothers. The sharp decline of consumer demand during and after the financial crisis, especially for durable goods, has been pegged as main driver of the trade collapse in 2008/2009 (e.g., Bems, Johnson, & Yi, 2013; Eaton, Kortum, Neiman, & Romalis, 2016). While our analysis can only draw on an early cutout of 2020 economic data and falls short of robustly identifying causal mechanisms, it offers several pieces of evidence that point towards COVID-19-related ramifications on both the demand and supply side. This simultaneity—as already argued by Baldwin (2020)—is likely a key feature that explains the sharper contraction of exports and imports than after the Lehman Brothers bankruptcy.

## Data on Swiss trade and the COVID-19 pandemic

This study builds on official trade data provided by the Swiss Federal Customs Administration (FCA). Swiss trade data is released at a high frequency, represents a significant share of Switzerland’s economic activity, and can be disaggregated across several dimensions including product groups or trading partners.

We combine weekly and monthly data on trade in goods, but exclude trade in services which is published on a quarterly basis and is generally subject to significant revisions. Weekly trade data has the advantage that it allows to track short-term fluctuations of economic activity with a delay of only a few days. Monthly data is published 2 weeks into the subsequent month, but in return allows for cleaner year-on-year comparisons. Moreover, reporting of weekly export data has not been standardized before February 2013, which precludes historical comparisons with weekly data previous to that date. Unless stated otherwise, we use nominal and seasonally unadjusted data.

Our analysis disaggregates trade data along trading partners and product groups. Our visual analysis mostly focuses on Switzerland’s top ten trading partners that account for 70% of Switzerland’s foreign trade in goods. Moreover, we use the FCA’s main product classification, which distinguishes broadly between twelve types of goods.^3^ Table 1 characterizes Swiss foreign goods trade along these two dimensions.

**Table 1:** Switzerland’s main trading partners and product groups

	Total trade		Exports		Imports	
	2019	2005	2019	2005	2019	2005
*Total (in bn CHF)*	*447.5*	*306.1*	*242.3*	*157.0*	*205.2*	*149.1*
*Total (in % of GDP)*	*63.9*	*60.1*	*34.6*	*30.9*	*29.3*	*29.3*
***Trading partner (share in %)***						
Germany	21.9	26.2	18.2	19.9	26.2	32.8
USA	12.5	7.6	17.3	10.4	6.7	4.7
Italy	7.3	10.1	5.8	9.2	9.1	11.0
France	6.6	9.3	5.9	8.6	7.4	10.0
China	6.3	2.2	5.5	2.1	7.3	2.3
UK	4.2	4.7	3.8	5.1	4.6	4.3
Austria	3.2	4.0	2.4	3.3	4.0	4.8
Spain	3.1	3.4	3.2	4.2	3.0	2.7
Japan	2.6	2.8	3.3	3.6	1.6	1.9
Netherlands	2.5	4.3	2.4	3.5	2.7	5.0
Other countries	29.9	25.5	32.1	30.1	27.4	20.7
***Product group (share in %)***						
06 - Pharmaceuticals	37.4	28.6	47.3	34.9	25.7	22.0
11 - Prec., watches, jewellery	18.1	12.6	20.8	17.6	14.8	7.2
09 - Machines	14.3	21.3	13.2	22.4	15.6	20.1
08 - Metals	6.4	7.9	5.6	7.4	7.3	8.3
10 - Vehicles	5.6	6.1	2.3	2.8	9.5	9.6
01 - Agriculture	5.5	5.3	4.2	3.3	7.1	7.4
03 - Textiles	3.8	4.3	2.1	2.7	5.8	5.9
02 - Energy	2.6	4.8	1.0	2.2	4.5	7.5
05 - Leather	2.6	3.1	1.9	2.7	3.4	3.5
12 - Various	1.6	2.2	0.5	1.2	2.8	3.3
04 - Paper	1.3	2.8	0.8	2.2	1.9	3.4
07 - Stones and earth	0.9	1.1	0.4	0.6	1.4	1.8

*Note*: All numbers refer to trade in goods excluding precious metals (mainly gold), gems, and other valuables. See Table 4 in the Appendix for the full definition of the product groups. *Source*: FCA

In 2019, imports totaled 205 billion CHF while exports amounted to 242 billion CHF.^4^ Between 2005 and 2019, exports have risen by 54.3%, and imports by 37.6%. With regard to the main trading partners, Switzerland is traditionally oriented towards the neighboring European Union. In the past 15 years, Swiss trade with the USA and China has grown disproportionally, especially on the export side. In 2019, most trade occurred with Germany (97.9 bn CHF, 21.9%), followed by the USA (12.4%), Italy (7.3%), France (6.6%), China (6.3%), and the UK (4.2%).

Concerning trade by product groups, we observe an increasing trade share of chemical and pharmaceutical exports: In 2005, chemical and pharmaceutical exports amounted to 54.8 billion CHF (34.9%), while they reached 114.6 billion CHF (47.3%) in 2019. Similarly, yet to a lesser extent, the trade share of precision instruments, watches, and jewelry also increased over the same period. The exports of this group are mainly driven by the exports of watches, while imports are largely dominated by jewelry. On the losing side, we find the products of the machinery and metal industry, a sector of the economy that was hit hard in the aftermath of the global financial crisis 2008 and has not yet fully recovered.

**Fig. 1:**
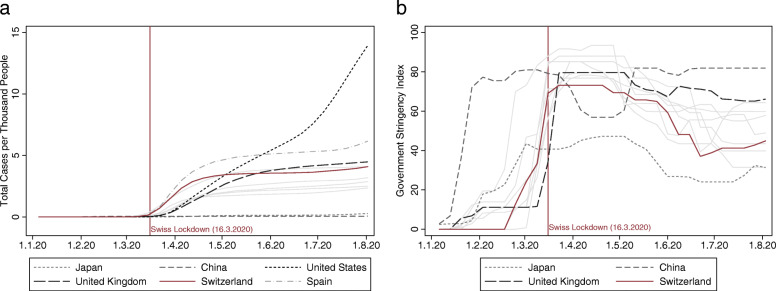
Total of COVID-19 cases and stringency of countermeasures. *Note*: Panel **a** plots the total number of COVID-19 cases per 1000 people for Switzerland and its ten main trading partners. Panel **b** plots the stringency of COVID-19 countermeasures by Switzerland and its ten main trading partners; higher values indicate more stringent measures. *Sources*: Johns Hopkins University’s Coronavirus Resource Center, Oxford University Coronavirus Government Response Tracker

We complement the trade data with information on the spread of COVID-19 across countries and its accompanying containment measures by governments. We draw data on the total number of cases per thousand inhabitants from *Johns Hopkins University’s Coronavirus Resource Center*. Panel a of Fig. 1 shows that lockdown measures in Switzerland were adopted when the COVID-19 case count was only 0.26 per 1000 inhabitants.^5^ Panel a of Fig. 1 further illustrates the quick spread of the disease and considerable differences in when and how the first wave of COVID-19 infections occurred. One explanation for the cross-country heterogeneity in the shape of the first wave of infections is the variation in both the measures that governments implemented and when they set them in place. As depicted in panel b of Fig. 1, *Oxford University’s Coronavirus Government Response Tracker* provides a country-specific measure ranging from 0 to 100 on how strongly governments intervened to contain the spread of the pandemic. Such measures include the shutdown of businesses, the closure of schools, and severe travel restrictions.

## Swiss foreign trade during the COVID-19 crisis

We now present the development of Swiss foreign trade during the COVID-19 crisis until summer 2020. First, we discuss weekly dynamics from an aggregate perspective in Section 3.1, and then turn to differences across trading partners and product groups in Section 3.2.

### Weekly trade dynamics

**Fig. 2:**
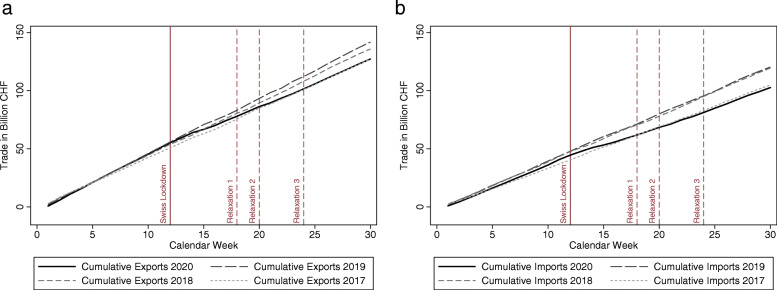
Cumulative exports and imports during the COVID-19 crisis compared to previous years. *Note*: Nominal and non-seasonally adjusted weekly data for Switzerland. *Relaxation 1*, reopening of personal care and gardening center; *Relaxation 2*, reopening of compulsory school, public transport, retail trade, and restaurants (partially); *Relaxation 3*, most remaining restrictions are lifted. *Source*: FCA

Figure 2 plots the cumulative nominal value of goods exported (panel a) or imported (panel b) by Switzerland in billion CHF during the first 30 calendar weeks of 2020. As a benchmark, we also display the weekly exports and imports of the three previous years, namely 2017 to 2019.

On March 16, that is in calendar week 12, the Swiss Federal Council declared an *Extraordinary Situation* for Switzerland invoking the Federal Epidemics Act. All shops, restaurants, bars, and leisure facilities had to remain closed until the gradual relaxation of mitigation measures in May and June. This event is labeled as “Swiss Lockdown” in Fig. 2. Prior to that date, exports and imports reached levels similar to those of 2019 and 2018 but clearly exceeded trade levels in 2017. The positive trade dynamics in January and February are consistent with the economic recovery at the international level which took place after the economic slowdown in the second half of 2019.

Figure 2 illustrates that the period shortly after the lockdown in March marks an inflection point: Both exports, shown in panel a, and imports, shown in panel b, begin to bend downwards around week 15. Following the introduction of the COVID-19 containment measures (which largely coincided with the global spread of the virus), Switzerland’s foreign trade in goods fell sharply: while weekly exports hovered around 5 billion CHF at the beginning of the year, they dropped by almost 25% to an average of 3.8 billion CHF during the most stringent phase of the lockdown. The shock had a similar impact on imports, which fell by about 30% compared to the pre-crisis level. Although a mild recovery of trade can be observed at the end of April, weekly trade volumes have remained at or even below trade volumes in 2017.

Between the start of the lockdown in week 12 and the third relaxation phase beginning in week 23, the trade collapse accumulated to 8.1 billion CHF in exports and 10.0 billion CHF in imports compared to 2019. At the end of the covered time period in week 30 (end of July), the accumulated loss since week 12 even amounted to 14.1 billion CHF in exports and 14.7 billion CHF in imports. While the gap in weekly trade levels has substantially narrowed, a full recovery of the cumulative trade volume to 2019 levels appears very unlikely.

### Heterogeneity across trading partners and product groups

**Fig. 3:**
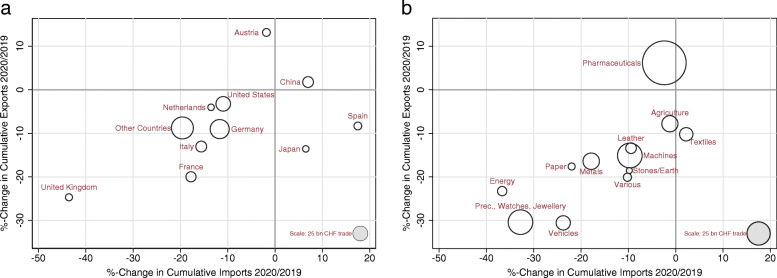
Cumulative exports and imports during the COVID-19 crisis by trading partners and product groups, first half of 2020. *Note*: Nominal and non-seasonally adjusted monthly data for Switzerland. The figures show the percentage change in cumulative exports (*y*-axis) and imports (*x*-axis) from January to June 2020 (i.e., calendar weeks 1 to 26) compared to the same period in 2019. Marker sizes indicate the relative trade volumes (exports + imports) in 2020. See Table 1 for details on the different trading partners and product groups. *Source*: FCA

The previous section documents that both aggregate exports and imports suffered substantial losses following the onset of the COVID-19 crisis. We now look at how the drop in Swiss foreign trade is distributed across different trading partners and product groups. Figure 3 summarizes the main findings by showing the cumulative change in exports (vertical axis) and imports (horizontal axis) during the first half year of 2020 compared to the same period of 2019. Panel a displays changes in trade by trading partners, while panel b plots changes across product groups. Marker sizes in both graphs indicate relative trade volumes (exports+imports) between January and June of 2020. A subplot-specific marker size corresponding to 25 billion CHF is shown in the lower right corner of each figure.^6^

Starting in the upper right quadrant of panel a, we observe that trade with only one country, namely China, increased in 2020 compared to 2019. The other main trading partner experiencing an increase in exports (but contraction in imports) is Austria. The rise in Japanese and Spanish imports came along with a fairly sharp decrease in their exports. The majority of countries are located in the left bottom quadrant, implying that both imports and exports decreased. In the case of the UK, exports declined by more than 25%, while imports fell by 40%. The neighboring countries Italy, Germany, and France show substantial losses in exports and imports ranging between 10 and 20%. Concerning the USA, exports only dropped marginally, while imports fell substantially.

When we disaggregate the change in trade flows by product group in panel b, the performance of chemical and pharmaceutical products stands out. As discussed in Section 2, the share of this product group has risen significantly in recent years and reached 37.4% of total trade in 2019. It is the only product group for which exports have risen in the first half of 2020 compared to 2019. In recent years, exports of this product group have grown by an average of 1% per month. Several factors explain the continued expansion of pharmaceuticals exports in 2020: *First*, foreign demand for Swiss pharmaceutical products is particularly inelastic with respect to economic and exchange rate shocks. These highly specialized products are typically protected by patents, which results in a lack of substitutes. *Second*, in times of crisis, people are more likely to reduce their consumption of durable goods (cars, appliances, etc.) than their health care spending. This is probably even more the case in times of a pandemic. On the import side, the only product group having increased during the COVID-19 crisis is textiles, clothing, and shoes. This can mainly be attributed to the sharp increase in demand for masks and protective clothing. The remaining product groups registered moderate to significant declines in both exports and imports. For instance, export-oriented manufacturing industries like machinery, electronic devices, and industrial metals registered substantial declines of more than 10% in cumulative exports. Symptomatically, trade in business cycle sensitive goods like precision instruments, jewelry, or vehicles dropped steepest between January and June 2020.

Overall, exports fell by 8.4% and imports dropped by 13.3% in the first half year of 2020 compared to 2019. If we consider exports and imports without chemical and pharmaceutical products, which proved much more resilient than other products during the first phase of the COVID-19 crisis, the trade plunge even amounted to 17.1% for exports and 21.4% for imports.

## Comparison with the global financial crisis

In order to put the trade collapse during the COVID-19 crisis into perspective, we compare the 2020 development to the drop in trade which occurred after the bankruptcy of Lehman Brothers in September 2008 and the subsequent recession.

Since World War II, global trade as a share of world GDP increased steadily. A heightened world trading potential, reductions in trade barriers, and greater vertical supply integration (among other factors) boosted the trade-to-GDP ratio from around 25% in 1960 to 60% in 2008.^7^ Then, however, the financial crisis and its consequences led to a decline in world trade of more than 10% and global trade as a share of GDP fell to 52.3% in 2009—the largest decline of global trade in decades (Baldwin, 2009; Baldwin & di Mauro, 2020). Moreover, the negative shock permanently reduced global trade’s long-term growth rate.

**Fig. 4:**
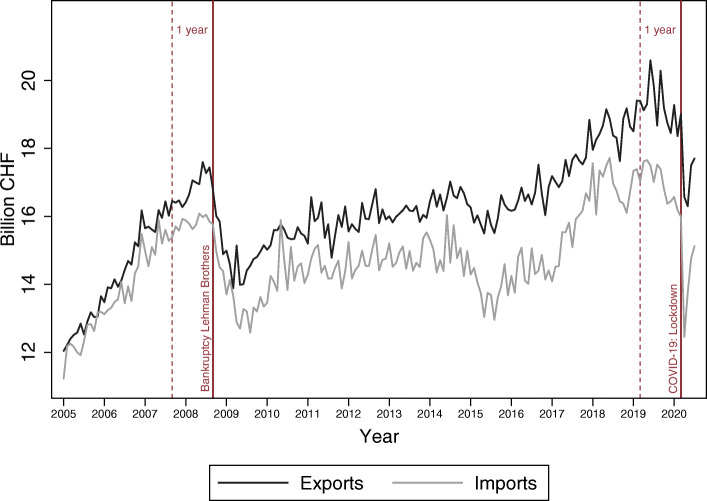
Monthly Swiss exports and imports since 2005. *Note*: Nominal and seasonally adjusted monthly data for Switzerland. *Source*: FCA

Figure 4 illustrates monthly, nominal and seasonally adjusted Swiss exports and imports since 2005. We observe a steep decline in both exports and imports after the bankruptcy of Lehman Brothers in September 2008. In the aftermath of the financial crisis, growth of Swiss foreign trade remained curbed for several years. A range of factors such as the appreciation of the Swiss Franc or the European sovereign debt crisis impeded a full recovery. Driven by dynamic foreign and domestic demand, exports and imports experienced another episode of high growth between 2016 and 2019. In the context of rising protectionism as well as slowing domestic demand, both global and Swiss trade reached a plateau in the second half of 2019.

**Fig. 5:**
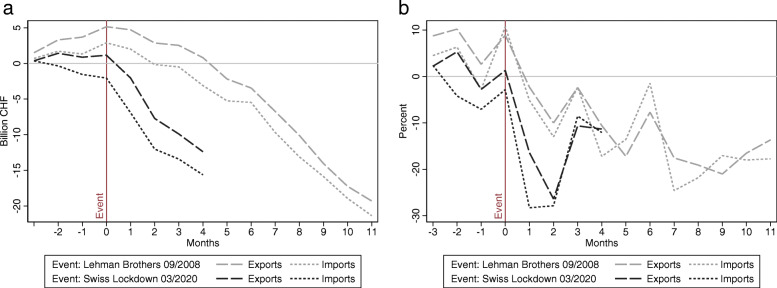
Financial crisis vs. COVID-19 pandemic: year-on-year trade changes. *Note*: Nominal and non-seasonally adjusted monthly data for Switzerland. The figures illustrate Switzerland’s changes in trade volumes following the bankruptcy of Lehman Brothers in September 2008 and the COVID-19 lockdown in March 2020. Panel **a** shows cumulative year-on-year absolute differences (beginning 3 months prior to the event), while panel **b** plots the year-on-year monthly growth rate in percent. *Source*: FCA

Figure 4 allows to get a first impression of the trade collapse in 2020 caused by COVID-19 compared to its decline during the financial crisis 2008/2009. In April 2020, Swiss exports fell to a level last reached in January 2016, and for imports, the downturn was even more pronounced. How does the recent decline in trade compare to the collapse during the financial crisis 2008/2009? To answer this question, we provide two plots in Fig. 5. For both time windows, we first define a specific event that triggered the deterioration of foreign goods trade: the bankruptcy of Lehman Brothers in September 2008 and the Swiss lockdown in mid-March 2020. Then, we calculate the cumulative trade volumes in panel a as well as the monthly percentage changes in panel b. As a reference period, we use the corresponding month of the previous year.

Panel a of Fig. 5 indicates that the 2020 trade collapse evolved much faster than in the aftermath of the Lehman Brothers bankruptcy. Within 4 months, both exports and imports fell by a cumulative sum of about 14 billion CHF. By contrast, it took about 9 months after the bankruptcy of Lehman Brothers in 2008 until the cumulative loss in exports and imports reached such levels. The data not only suggests, however, that the COVID-19-induced collapse was more rapid, but that the recovery could be faster, too. As panel b of Fig. 5 shows, exports exhibited a negative growth trend for 9 months and imports started to recover within 7 months after the 2008 event. During the COVID-19 crisis, the recovery and stabilization of imports and exports already commenced in the third month after the lockdown in mid-March.

We can dive further into the details by again looking at differences across trading partners and product groups. For the majority of Switzerland’s main trading partners, the decline in cumulative exports and imports during the current crisis was greater than during the global financial crisis (data not shown). Extreme cases are Japan and the UK: While Swiss imports from Japan evolved similarly in 2020 and 2008/2009, the drop in exports between March and July 2020 exceeded the contraction between September 2008 and January 2009 by 40 percentage points. Concerning Great Britain, the dip in Swiss exports and imports during COVID-19 was more than 20 percentage points deeper compared to the first months of the financial crisis. The magnitudes are smaller for France, Italy, Germany, and the USA, but losses are still considerably larger in 2020 than during the global financial crisis 2008/2009. On the other end of the ranking, we find China, where both exports (+ 7 percentage points) and imports (+ 18 percentage points) performed much better during the 2020 crisis than in 2008/2009. China’s relative trade statistics are trailed by those for Spain and Austria, the only other major trading partners of Switzerland performing better during the COVID-19 crisis than in the aftermath of the Lehman Brothers bankruptcy.

When we compare the trade statistics between the two trade collapses disaggregated by product groups, we observe the greatest gap in the groups “precision instruments, watches and jewelry” as well as “vehicles.” Considering that the former group accounts for about 18% in Swiss trade, additional losses in this category (about a 40 percentage points stronger drop in 2020 than 2008) weigh heavily on aggregate dynamics of exports and imports. On the upside, trade volumes in the largest product group, namely “chemicals and pharmaceuticals,” have been equivalently resilient to negative shocks during both crises.

## What explains the Swiss trade collapse in 2020?

Even when compared with other major events such as the financial crisis of 2008, the contraction witnessed in spring 2020 is unprecedented. We now discuss potential drivers for this rapid decline. In particular, we examine and discuss the following channels: (i) COVID-19 induced demand shocks, (ii) COVID-19 induced supply shocks, (iii) protective trade measures due to COVID-19, and (iv) exchange rate movements due to major shifts in currency demand.

### COVID-19 induced demand shocks

We begin the discussion with another appraisal of the product-specific change in trade volumes plotted in panel b of Fig. 3. The two product groups that suffered the largest losses in the first two quarters of 2020 are “precision instruments, watches and jewelry” and “vehicles.” Both groups primarily comprise durable consumption goods, such as watches and passenger cars. This suggests that a contraction in Swiss and foreign demand is likely a major driver behind the trade collapse.

The Swiss watch industry suffered particularly in the context of the current crisis: the sudden stop in international tourism activities, combined with the temporary closures of retail stores, brought domestic and foreign sales to a near standstill. Concerning the deterioration in the trade of vehicles, it is essentially attributable to the contraction in domestic demand for passenger cars, causing imports of vehicles to collapse both in Switzerland and abroad.

Similar, albeit less pronounced patterns were documented for trade in the aftermath of the global financial crisis: According to Eaton, Kortum, Neiman, & Romalis, (2016), plunging demand was the driving force behind the considerable trade contraction during and after the financial crisis of 2008. In general, this explanation fits well with the idea that economic uncertainty causes consumers to defer spending, especially on non-essential and expensive products.^8^

**Fig. 6:**
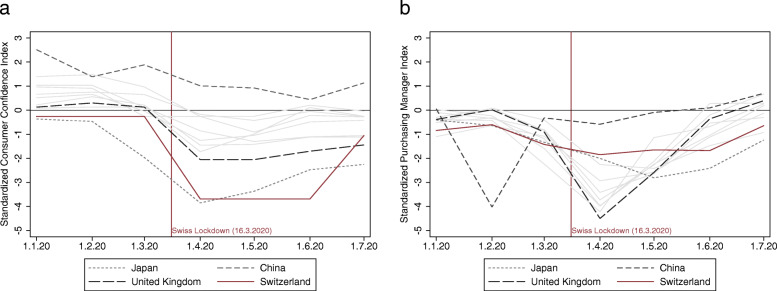
Standardized consumer and producer confidence in 2020. *Note*: Panel **a** plots the consumer confidence index for Switzerland and its ten main trading partners. Panel **b** shows the Manufacturing Purchasing Manager Index (PMI) for the same sample. All series are standardized by deducting the long-run average and dividing the demeaned series with the long-run standard deviation. *Sources*: IHS Markit, Macrobond

To further assess the link between contraction of demand and declining trade flows, we plot standardized consumer confidence indices for Switzerland and its main trading partners in panel a of Fig. 6.^9^ While consumers in most countries were relatively optimistic at the outset of 2020, the spread of COVID-19 led to a substantial drop in consumer confidence of about 2 standard deviations (SD) around March. All countries plotted in Panel (a) of Fig. 6 experienced a drop in consumer confidence, but the slump is most pronounced in Japan, Switzerland, and the UK. This pattern also fits with the trade dynamics discussed in Section 3: The plunge in Swiss exports to the UK and Japan in the first half of 2020 was disproportionately deep, and foreign exports to Switzerland—which are decisively driven by Swiss demand—fell even more sharply than Swiss exports to other countries.

We further assess the link between consumer confidence and Swiss exports in columns (1) and (6) of Table 2. The sample includes all Swiss trading partners that exceed a minimum trade level and where data on the variables of interest is available.^10^ All estimations include time period fixed effects that absorb aggregate time trends, so that the correlations reflect cross-sectional heterogeneity and country-specific fluctuations over time. The models based on monthly frequency (in column 1) and quarterly frequency (in column 6) show that consumer confidence in the importing countries (plus time period fixed effects) explain 14 to 24% in the variation across country-specific growth rates of Swiss exports in the first 6 months of 2020. The correlation is not only robust to the use of different data frequencies (monthly vs. quarterly), but also quantitatively substantial: A one-standard deviation drop in an importer country’s consumer confidence is roughly associated with a 5 percentage point decline in Swiss exports to that country.

**Table 2:** Correlates of Swiss exports between January and June 2020

% Change in exports compared to 2019	Monthly data					Quarterly data				
	(1)	(2)	(3)	(4)	(5)	(6)	(7)	(8)	(9)	(10)
*Consumer*	4.57 ^∗^				4.33 ^+^	5.31 ^∗^				5.47 ^∗∗^
*Confidence (in SD)*	(2.15)				(2.20)	(2.51)				(2.57)
Covid-19 *cases*		− 2.21 ^∗∗^		− 2.23 ^∗∗^	− 2.56		− 0.89 ^∗∗^		− 0.86 ^∗∗^	− 0.22
*per 1000 people*		(0.74)		(0.83)	(2.18)		(0.28)		(0.30)	(1.13)
*Stringency index*			− 0.02	0.02	− 0.07			− 0.10	− 0.08	− 0.22
*countermeasures*			(0.15)	(0.15)	(0.16)			(0.15)	(0.16)	(0.15)
*R*^2^	0.14	0.10	0.09	0.10	0.15	0.24	0.17	0.15	0.17	0.25
Trading partners	40	55	55	55	40	40	55	55	55	40
Observations	215	300	300	300	215	79	110	110	110	79

*Notes:* The dependent variable is the percentage change in monthly (col. 1–5) or quarterly (col. 6–10) exports compared to 2019. All models include *time period fixed effects*. Covid-19 *cases per 1000 people* denotes the increase in a country’s confirmed Covid-19 cases per 1000 inhabitants during that month/quarter. *Stringency index countermeasures* represents the average stringency score of a country during that month/quarter taking values between 0 (no measures) and 100 (maximum stringency). *Consumer confidence* represents the standardized monthly/quarterly deviation from the long-term mean in a country’s consumer confidence. We restrict the sample to countries with a minimum annual trade value of 500 million CHF and monthly trade values of at least 25 million CHF in 2019. Descriptive statistics are reported in Table 5 in the Appendix. Standard errors (in parentheses) are clustered by trading partners. ^+^*p* < 0.10, *p < 0.05, **p < 0.01

**Table 3:** Correlates of Swiss import flows between January and June 2020

% Change in imports compared to 2019	Monthly data					Quarterly data				
	(1)	(2)	(3)	(4)	(5)	(6)	(7)	(8)	(9)	(10)
*Purchasing Manager*	4.62 ^+^				4.22	6.93				6.67
*Index (in SD)*	(2.65)				(2.80)	(4.60)				(4.66)
Covid-19 *cases*		− 2.08		− 1.55	− 1.55		− 1.08 ^+^		− 0.84	− 2.65
*per 1000 people*		(1.38)		(1.40)	(6.58)		(0.62)		(0.60)	(2.86)
*Stringency index*			− 0.33 ^∗^	− 0.30 ^∗^	− 0.12			− 0.49 ^∗^	− 0.45 ^∗^	− 0.15
*countermeasures*			(0.12)	(0.12)	(0.13)			(0.20)	(0.19)	(0.17)
*R*^2^	0.14	0.09	0.10	0.10	0.15	0.11	0.05	0.07	0.08	0.15
Trading partners	28	55	55	55	28	28	55	55	55	28
Observations	164	300	300	300	164	56	110	110	110	56

*Notes:* The dependent variable is the percentage change in monthly (col. 1–5) or quarterly (col. 6–10) imports compared to 2019. All models include *time period fixed effects*. Covid-19 *cases per 1000 people* denotes the increase in a country’s confirmed Covid-19 cases per 1000 inhabitants during that month/quarter. *Stringency index countermeasures* represents the average stringency score of a country during that month/quarter taking values between 0 (no measures) and 100 (maximum stringency). The *Purchasing Manager Index* represents the monthly/quarterly standardized deviation from the long-term mean in managers’ confidence. We restrict the sample to countries with a minimum annual trade value of 500 million CHF and a monthly trade value of at least 25 million CHF in 2019. Descriptive statistics are reported in Table 5 in the Appendix. Standard errors (in parentheses) are clustered by trading partners. ^+^*p* <0.10, *p <0.05, **p <0.01

A central question is whether differences in COVID-19 infections and/or containment measures across trading partners (see Fig. 1) explain differences in Swiss export dynamics. If one or both of these two COVID-19 induced shocks to foreign demand play a key role in the trade collapse of 2020, we should see that exports declined more strongly when being shipped to trading partners particularly affected by COVID-19. To test this hypothesis, Table 2 presents the estimates for six regressions models that provide correlations between our two main COVID-19 measures presented in Fig. 1 with year-on-year changes in Swiss exports between January and June 2020. Columns (2) to (4) of Table 2 use monthly data, while columns (7) to (9) re-estimate the same models based on quarterly data.

The results unambiguously suggest that differences in COVID-19 infection rates help explain changes in country-specific exports. Specifically, the larger the number of COVID-19 cases per 1000 people in a partner country, the larger the decline in Swiss exports to this country. At the same time, we find virtually zero correlation between the stringency of COVID-19 containment measures and export changes. Apparently, demand for Swiss exports was primarily driven by the spread of COVID-19 in trading partner countries, but not by the stringency of their countermeasures.

Finally, we examine the hypothesis that the impact of COVID-19 for Swiss exports runs via its adverse effect on consumer confidence in importing countries. If that is the case, we would expect that the correlation between the two COVID-19 measures and Swiss exports weakens while the estimate for consumer confidence remains stable when these three variables are jointly included in the regression model. Indeed, the point estimates for consumer confidence in columns (5) and (10) remain virtually unchanged, while the estimate for COVID-19 cases per 1000 people becomes insignificant. Moreover, regressing consumer confidence on COVID-19 cases per 1000 people yields a strong negative relationship (results not shown) confirming that COVID-19 cases lower consumer confidence and with it aggregate demand.^11^

To assess the robustness of these results, we vary the model specifications for our analysis of monthly trade data along three dimensions (results not shown): *First*, we extend the regression model with monthly *exchange rates*, which hardly affects the point estimates for our variables of interest. *Second*, we add *trading partner fixed effects* that account for time-constant country characteristics that may lead to spurious correlations. For instance, the quality of public health policies (e.g., testing regimes) likely differ across countries, and these differences may be correlated with the long-term trade composition. While the results for both COVID-19 measures remain qualitatively unaltered, the point estimates for consumer confidence drop by about 20% and become insignificant (*t*-value = 1.2–1.4).^12^*Third*, we vary the *trade threshold* that we apply to eliminate extreme outliers. Overall, the main insights reported in Table 2 are robust to reasonable changes in this threshold.

In summary, the available evidence suggests that a demand side contraction driven by the global spread of COVID-19 was a major ingredient leading to the unprecedented trade collapse in the first half of 2020. We next discuss to what extent supply side dynamics explain the observed patterns.

### COVID-19 induced supply shocks

Another likely channel are contractions on the supply side, as containment measures imposed by governments complicated business operations, or because employees missed work (Koren & Pető, 2020). Although the drop in intermediate and capital goods was not as pronounced as for consumer durable goods, panel b of Fig. 3 shows that products of the groups “Machines, appliances, electronics” and “Metals” were traded considerably less in 2020 than in 2019.

Capacity utilization in the Swiss mechanical and electrical engineering industries fell far below its long-term average and companies complained about high obstacles in production due to the COVID-19 restrictions. To examine the link between business restrictions and Swiss foreign trade in 2020, we plot standardized manufacturing Purchasing Manager Indices (PMI) for Switzerland and its main trading partners in panel b of Fig. 6.^13^ These series capture the managers’ sentiments about the general business environment, and hence partially measure whether producers face (cost-driving) obstacles in their daily operations.

While producer sentiment in early 2020 was slightly below the long-run average, the spread of COVID-19 led to a very pronounced drop of about three standard deviations around March. China, where the virus occurred first, run about 1 month ahead of the other countries and recovered quickly. The UK suffered from the deepest plunge in producer sentiment, while Switzerland and Japan—quite in contrast to the consumer confidence series—experienced fairly contained fluctuations in their PMIs. It is also noteworthy that producer confidence, despite the deeper drop, recovered more quickly than consumer sentiment.

Total Swiss imports in the first half of 2020 fell by 13.3% compared with 2019. However, imports of intermediate products decreased by 16.9%. Intermediate goods account for a large and growing share of international trade due to global value chains. Switzerland as a high-wage country relies heavily on such intermediate goods from abroad. They account for more than one-fifth of all imports.

If the COVID-19-induced shock to foreign production plays a key role in the trade collapse of 2020, we should see that imports declined more strongly when coming from trading partners particularly affected by the pandemic. To test this hypothesis, Table 3 emulates our previous analysis on exports and regresses the percent change in Swiss imports (in the first half of 2020 compared to 2019) on time period fixed effects and three explanatory variables of interest: the number of COVID-19 cases, the stringency index, and the PMI.

The estimation results suggest that—in contrast to the results on exports—variation in the stringency of government-imposed containment measures are more consistently correlated with year-on-year changes in Swiss imports. Both monthly and quarterly data show that stricter government restrictions in foreign countries were associated with sharper declines in Swiss imports from those countries (see columns 3 and 4 and 8 and 9). Like for exports, the number of confirmed COVID-19 cases is also negatively correlated with import growth; while the point estimates remain similar in magnitude, they are less precisely estimated with imports so that three out of four coefficients in columns (2), (4), (7), and (9) are statistically insignificant.

Again, there is some evidence supporting the narrative that an important channel of the COVID-19 impact runs via the confidence of economic agents, although the very small sample size (i.e., 28 countries with PMI data) handicaps this analysis. Columns (1) and (6) of Table 3 shows that Swiss imports from countries with low PMI scores dropped particularly strongly (*t*-values, 1.5–1.7). Moreover, the point estimates for countermeasure stringency drop by around 60% once we include the PMI in columns (5) and (10), while the PMI’s coefficient decreases only slightly and is a bit less precisely estimated (*t*-values = 1.4–1.5). Regressing the monthly PMI on the government stringency index further confirms that the government measures dragged down producer sentiments (*t*-value, − 4.9, results not reported).

In summary, this analysis confirms the hypothesis that the spread of COVID-19 negatively impacted international trade by affecting both the demand and the supply side. While the data suggest that foreign demand for Swiss goods was almost exclusively driven by confirmed COVID-19 cases, the foreign supply of goods is more strongly correlated with the stringency of government measures. If assessed jointly (results not shown), namely by modeling the value of total trade instead of imports in columns (4) and (9), the negative correlation with confirmed COVID-19 cases (*t*-value for monthly data, − 2.6; *t*-value for quarterly data, − 2.4) clearly dominates the correlation with public health measures (*t*-value for monthly data, − 1.3; *t*-value for quarterly data, − 1.7). Overall, the data lends little support to the narrative that the costly economic fall-out of COVID-19 should be *primarily* attributed to the unprecedented public health policies; yet, we find some evidence that stringent containment measures adopted by trading partners imposed costly barriers to the foreign producers of Swiss imports.

In the following, we assess two additional channels, which might help to explain the contraction of Swiss trade in 2020: protective trade policies and exchange rate shifts.

### Protectionism

**Fig. 7:**
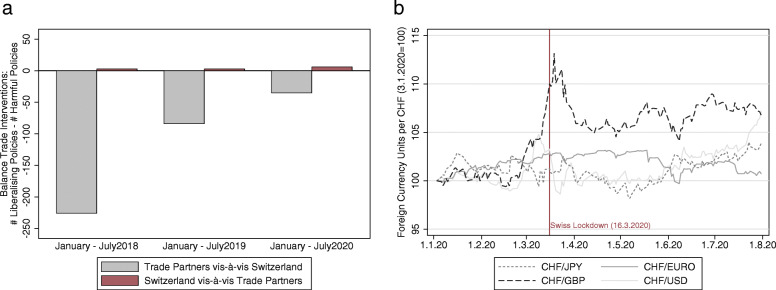
Global trade interventions and Swiss Franc against major currencies. *Note*: Panel **a** illustrates global trade interventions for 2018, 2019, and 2020; a positive value indicates that the number of newly imposed liberalizing interventions exceeded the number of newly imposed harmful interventions, while a negative value implies that trade policy became more protectionist. Panel **b** plots the CHF/JPY, CHF/EUR, CHF/USD, and CHF/GBP exchange rates normalized to 100 on the first trading day in 2020, i.e., 3.1.2020; a rise in the index value from 100 to 105 indicates a 5 percent appreciation of the Swiss franc. *Sources*: Global Trade Alert https://www.globaltradealert.org/, Swiss National Bank

Throughout the COVID-19 pandemic, countries around the world erected new barriers for travel and trade in an effort to contain the virus. Concerning goods trade, some countries also imposed protective restrictions on exports of highly essential products, such as pharmaceuticals and food. A systematic look at global trade measures, however, makes protective trade policies a very unlikely driver behind the documented trade collapse. Neither Switzerland nor its main trading partners erected an unusually high number of protective trade barriers. The trend between January and July points rather to the contrary, as *Global Trade Alert* data (see Evenett and Fritz (2020)) plotted in panel a of Fig. 7 documents: The number of protective trade measures relative to the number of liberalizing policies was much higher in 2018 and 2019 than in 2020. In the first half of 2020, 156 new harmful trade restrictions by trading partners vis-à-vis Switzerland exceeded 121 liberalizing policies by a total of 35. This is substantially less than in the previous 2 years with a balance of − 226 in 2018 and − 84 in 2019.

### Exchange rate movements

The Swiss franc is well known to serve as a safe haven currency during times of global economic uncertainty (e.g., Jaeggi, Schlegel, & Zanetti, 2019). We therefore briefly address, whether COVID-19 related uncertainty led to major inflows of capital therewith appreciating the Swiss franc and putting pressure on the export-oriented industry; this was the case during the European debt crisis, culminating in the announcement of a minimum exchange rate floor by the Swiss National Bank in September 2011. We abstain from analyzing actual capital flows (which might have been neutralized by the Swiss National Bank), but instead discuss fluctuations in four major exchange rates during the period January to July 2020.

For three out of four major exchange rates plotted in panel b of Fig. 7 (namely, CHF/USD, CHF/EURO, CHF/JPY), the fluctuations in the first half of 2020 were limited to a narrow index-band spanning 95 to 105.^14^ Considering that the short-run exchange rate elasticity for Switzerland’s exports ranges from − 0.2 to − 0.6 (e.g., Hanslin, Lein, & Schmidt, 2016), these minor fluctuations can be safely ruled out as driving factors behind the documented trade collapse starting in mid-March. The main exception is the Swiss franc to British pound exchange rate that appreciated by 15% in early March. While this appreciation, together with Britain’s withdrawal from the EU on 31 January 2020, could have significantly contributed to the substantial shifts observed in Swiss-British trade, the overall exchange rate fluctuations were certainly too small to explain a relevant share of Switzerland’s unprecedented trade contraction in spring 2020. In fact, adding exchange rates to our models of monthly trade reported in Tables 2 and 3 leads to small (and insignificant) elasticity estimates of about − 0.2 for exports and 0.1 for imports without altering the results for our measures of COVID-19, consumer confidence, and producer confidence.

## Conclusion

The Swiss economy is deeply integrated in global value chains. Due to its detailed trade data that is published at a high frequency, Switzerland serves us as valuable case study for an early appraisal of trade dynamics during the ongoing COVID-19 pandemic. Using weekly and monthly trade data, we document how fast, to what extent, and along which dimensions the Swiss trade collapse evolved between January and July 2020.

Between the lockdown in mid-March and the end of July, the Swiss economy experienced trade losses of 14 billion CHF in exports and 15 billion CHF in imports compared to 2019. Product diversity potentially helped to prevent even greater losses: goods from the chemical and pharmaceutical industry were notably resilient, while all other sectors experienced dramatic declines in both imports and exports.

Our analysis of country-specific trade data suggests that the COVID-19-related losses can be attributed to both the spread of the pandemic as well as the contingency measures implemented by governments across the globe. The contraction in Swiss exports is correlated with the number of confirmed COVID-19 cases in importing countries, while Swiss imports are more strongly associated with the stringency of government measures in the exporting economy.

## Appendix

**Table 4:** Product classifications in the Swiss dataset

01	Forestry and agricultural products, fisheries
01.1	Food, beverages, and tobacco
01.2	Feeding stuffs for animals
01.3	Live animals
01.4	Horticultural products
01.5	Forestry products (not firewood)
01.6	Products for commercial/industrial processing such as oils, fats, plants and vegetable parts, etc.
02	Energy source
02.1	Solid combustibles
02.2	Petroleum and distillates
02.3	Gas
02.4	Electrical energy
03	Textiles, clothing, shoes
03.1	Textiles
03.2	Articles of apparel and clothing
03.3	Shoes, parts, and accessories
04	Paper, articles of paper, and products of the printing industry
04.1	Basic materials for paper production, cellulose (fiber), and paper and carton waste
04.2	Paper and carton in rolls, strips, or sheets
04.3	Goods from paper or carton
04.4	Products of the printing industry
05	Leather, rubber, plastics
05.1	Leather
05.2	Rubber
05.3	Plastics
06	Products of the chemical and pharmaceutical industry
06.1	Chemical raw materials, basic materials, and unformed plastics
06.2	Chemical end products, vitamins, diagnostic products, including active substances
07	Stones and earth
07.1	Mineral raw materials and basic products
07.2	Goods from stone and cement
07.3	Ceramic wares
07.4	Glass
08	Metals
08.1	Iron and steel
08.2	Non-ferrous metals
08.3	Metal goods
09	Machines, appliances, electronics
09.1	Industrial machinery
09.2	Agricultural machines
09.3	Household appliances
09.4	Office machines
09.5	Electrical and electronic industry appliances and devices
09.6	Military equipment
10	Vehicles
10.1	Road vehicles
10.2	Railed vehicles
10.3	Air- and spacecraft
10.4	Watercraft
11	Precision instruments, clocks and watches, and jewellery
11.1	Precision instruments and equipment
11.2	Watches
11.3	Jewellery and household goods made from precious metals
12	Various goods such as music instruments, home furnishings, toys, sports equipment, etc.
12.1	Exposed film
12.2	Music instruments
12.3	Home furnishings
12.4	Toys and sports equipment
12.5	Stationery goods
12.6	Various goods such as umbrellas, neon signs, festive articles, brushes, lighters, pipes, etc.

*Note*: The table shows all product groups of our data set. The classification follows the FCA’s grouping by the nature of goods

**Table 5:** Descriptives statistics

**Monthly data, Jan.–Jun. 2020**	Number	Mean	SD	Min	Max
*Year-on-year % changes exports*	300	− 3.49	35.02	− 70.70	309.21
*Year-on-Year % changes imports*	300	− 8.49	35.75	− 99.62	198.94
Covid-19 *cases per 1000 people*	300	0.52	1.36	0.00	14.80
*Stringency index countermeasures*	300	49.96	30.46	0.00	100
*Consumer confidence (in SD)*	215	− 0.69	1.30	− 3.85	2.51
*Purchasing Manager Index (in SD)*	164	− 1.62	1.77	− 7.39	1.37
**Quarterly data, Q1 and Q2 2020**	Number	Mean	SD	Min	Max
*Year-on-year % changes exports*	110	− 3.49	23.75	− 54.24	100.14
*Year-on-year % changes imports*	110	− 9.50	29.82	− 95.96	107.58
Covid-19 *cases per 1000 people*	110	1.41	3.44	0.00	32.77
*Stringency index countermeasures*	110	45.85	27.511	6.17	92.59
*Consumer confidence (in SD)*	79	− 0.62	1.23	− 3.38	1.93
*Purchasing Manager Index (in SD)*	56	− 1.59	1.27	− 5.19	0.30

*Note*: This table reports summary statistics for the variables used in the regression analysis reported in Tables 2 and 3. *Sources*: FCA, Johns Hopkins University’s Coronavirus Resource Center, Oxford University Coronavirus Government Response Tracker, IHS Markit, Macrobond

## Data Availability

The datasets generated and/or analyzed during the current study are available in the Harvard Dataverse repository, 10.7910/DVN/DDTCB.

## Abbreviations

Covid-19: Coronavirus disease 2019
PMI: Purchasing Manager Indices
SD: Standard deviation
FCA: Swiss Federal Customs Administration
CHF: Swiss Francs

## Footnotes

1 The Covid-19 pandemic is a global pandemic caused by severe acute respiratory syndrome coronavirus 2 (SARS-CoV-2). The outbreak was first detected in December 2019 in the Chinese city of Wuhan. The World Health Organization declared the outbreak a *public health emergency of international concern* on 30 January 2020, and a *pandemic* on 11 March. At the beginning of September 2020, more than 26 million cases of Covid-19 had been reported in more than 188 countries and territories, resulting in about 860,000 deaths.

2 Several platforms and special issues were launched to quickly disseminate scholarly work on the Covid-19 pandemic, including among others by the *Centre for Economic Research* (Link), the *European Economic Association* (Link), and the *Swiss Journal of Economics and Statistics* (Link).

3 For means of interpretation, we follow the classification by nature/type that is used within the national accounts. See Appendix: Table 4 for an overview of all product groups and https://www.ezv.admin.ch/ezv/en/home/topics/swiss-foreign-trade-statistics/daten/waren.htmlfor more detail on the different classifications.

4 All trade data used in this paper exclude valuables, i.e., precious metals (mainly gold), precious stones and gems, works of art, and antiques. These goods are excluded from the analysis because they are highly volatile, are quantitatively large, and contain no business cycle relevant information.

5 On March 15, Switzerland recorded a total of 2196 Covid-19 cases and a population of about 8.5 million. At that time, total cases per thousand people in China (0.17), the USA (0.01), and other countries were also fairly low but quickly growing.

6 We decided to cover the January to June (instead of July) window for two reasons: *First*, the period between January and June marks the first half year of 2020. *Second*, we use both quarterly data and monthly data in the regression analysis presented in Section 5, which therefore has to rely on the January to June window. In any case, adding trade data from July would not change the qualitative insights from Fig. 3.

7 See for instance Anderson and Van Wincoop (2004), Clemens and Williamson (2004), Freund (2009), Irwin (2002), or Jacks and Pendakur (2010) among others.

8 Brown, Fengler, Lalive, Rohrkemper, and Spycher (2020) document Swiss consumer spending during the Covid-19 crisis.

9 We retrieve consumer confidence series from the financial data provider *Macrobond* who compiles data from different national sources. Consumer confidence is measured via forward-looking questions asked to private households, such as *How do you think the general economic situation will develop over the next 12 months?*. While the consumer confidence series are available for a broad set of countries and follow standardized methods (e.g., European Commission, 2020), one caveat concerns unequal periodicity of the available series. In most countries, for instance in Germany, China, or the UK, consumer confidence data is available on a monthly basis, while for other countries, such as Switzerland and Japan, it is available on a quarterly basis. The lower frequency of the Swiss consumer confidence data hampers its comparability, yet higher frequency measures of consumer sentiment based on Google searches also confirm that Swiss consumer confidence took a deep dive in Spring 2020 and has not fully recovered in August (see Eichenauer, Indergand, Martinez, & Sax (2020)).

10 We restrict the estimation sample to those countries with a minimum annual total trade value of 500 million CHF and a monthly trade value of at least 25 million CHF in 2019, and apply this threshold to all estimations in Table 2. The aim of this procedure is to eliminate extreme outliers that can be traced back to periodically tiny trade volumes of small trading partners in the base year 2019. Since such potentially tiny numbers enter the denominator, even a small absolute increase in trade flows can lead to a relative increase of several thousand percentage points. We will also apply the same threshold to analyze imports in Table 3 of the following section; since Table 3 features PMI data as explanatory variable, where less than 30 countries are available, we chose a threshold that maximizes the number of countries with year-over-year trade changes in a low three digit region or below.

11 The *t*-value for the correlation between monthly Covid-19 cases and consumer confidence is − 1.4 in a model with month fixed effects and − 4.4 in a model without month fixed effects.

12 Note, however, that including trading partner fixed effects makes estimations vulnerable to inaccuracies in the timing, since the fixed effects model identifies the estimates based on within-group variation only. This may be an issue regarding consumer confidence data, which is only available at quarterly (not monthly) periodicity for several countries.

13 We retrieve PMI series calculated by *IHS Markit* via the financial data provider *Macrobond*. The PMI index summarizes the assessment of purchasing managers in the manufacturing sector concerning their current production, order backlog, and future business conditions. One challenge in the analysis of this index is its relatively narrow availability restricting our sample to only 28 trading partners of Switzerland.

14 The fairly mild fluctuations could at least partially be the result of interventions by the Swiss National Bank, as their foreign currency investments increased from 794 to 863 billion CHF during the discussed time period.
